# Public transport: the missing link in tuberculosis control in Somalia

**DOI:** 10.1016/j.jctube.2026.100635

**Published:** 2026-07-18

**Authors:** Mohamed Abdulkadir Hussein, Ilyas Abdullahi Khalif, Saida Abdirzak Mohamed, Sharmake Gaiye Bashir, Ahmed Abdinasir Abdulle

**Affiliations:** a Faculty of Health Sciences and Tropical Medicine, Somali National University, Mogadishu, Somalia; b Center for Health Research and Innovation, Somali National University, Mogadishu, Somalia; c Banadir Research, Consultancy & Evaluation Center, Mogadishu, Somalia; d Faculty of Health Sciences, Salaam University, Mogadishu, Somalia

**Keywords:** Tuberculosis, Public transport, Somalia, Airborne transmission

## Abstract

Tuberculosis (TB) remains a major public health challenge in Somalia, where conflict, displacement, delayed diagnosis, and fragile healthcare systems sustain ongoing transmission. While existing TB control efforts largely emphasize household and healthcare-associated exposure, the role of overcrowded public transport systems has received limited attention. In rapidly urbanizing Somali cities, particularly Mogadishu, minibuses and shared taxis represent enclosed, poorly ventilated, high-turnover environments that may facilitate airborne transmission of *Mycobacterium tuberculosis*. Evidence from high-burden settings demonstrates that public transport can function as a significant transmission setting because of prolonged close contact and inadequate airflow. Somalia's low case detection rates and frequent delays in treatment initiation further increase the likelihood that infectious individuals continue using crowded transport networks during active disease. This letter argues that public transport should be recognized as a neglected but modifiable determinant of TB transmission in Somalia. Integrating transport-focused interventions into national TB strategies, including awareness campaigns, community-based active case finding, improved ventilation practices, and mobility-informed surveillance, could provide a practical and context-appropriate opportunity to reduce transmission. Addressing public transport as a mobile transmission environment may strengthen TB control efforts and contribute to reducing the burden of disease in Somalia's fragile urban settings.

Dear editor,

Tuberculosis remains a leading cause of infectious disease mortality globally, with progress toward elimination slowed and, in some regions, reversed [1]. In Somalia, TB incidence and mortality are among the highest worldwide, compounded by conflict, displacement, and a fragile health system [2], [3]. While policy attention has focused on clinical and household transmission, everyday journeys in overcrowded public transport have been largely ignored. This letter aims to highlight public transport in Somalia particularly in Mogadishu and other urban centers as a missing but addressable link in TB control.

Airborne TB transmission is driven by prolonged exposure to infectious aerosols in enclosed, crowded, and poorly ventilated spaces. Studies from South Africa and other settings show that minibus taxis, trains, and buses can have higher estimated TB transmission risk than many workplaces or shops, even when some natural ventilation exists, because of high occupancy and turnover of passengers [1]. Experimental work on buses demonstrates that ventilation systems and airflow patterns can distribute infectious aerosols throughout the vehicle, while opening windows and doors and using masks markedly reduces exposure [4].

Somalia's context amplifies these risks. Low detection and delayed diagnosis mean many people travel while infectious, with advanced radiological disease common in Mogadishu, indicating prolonged, untreated transmission [3]. Weak surveillance and disrupted services in a conflict-affected health system further obscure where transmission occurs and hinder timely response [2]. Rapid urbanization and dense mobility patterns, especially in Mogadishu and other hubs, create recurrent crowding in minibuses and shared taxis. These vehicles, constantly circulating between settlements, markets and health facilities, function as mobile transmission hotspots: dynamic micro-environments where undiagnosed TB patients repeatedly mix with new susceptible passengers, seeding infection across neighborhoods. Similar patterns of high TB risk in public transport and other congregate settings have been documented in South Africa, Peru and Tanzania, underscoring their importance in high-burden, low-resource contexts [1], [4].

Recognizing Somali public transport as a TB determinant has immediate, feasible implications. First, targeted public awareness campaigns should use transport hubs and vehicles to communicate TB symptoms, cough etiquette, and the importance of early care-seeking, prioritizing urban centers with heavy commuter flows [2], [3]. Second, community-based active case-finding shown to increase detection when delivered with high coverage could be adapted to transport corridors and terminals, using mobile teams for symptom screening and sputum collection [5]. Third, low-cost ventilation measures, informed by evidence from minibus taxi studies, should be promoted: standardized window-opening practices, avoidance of fully closed vehicles when crowded, and, where feasible, simple structural modifications to improve airflow [1], [4]. Fourth, TB surveillance in Somalia should explicitly map and monitor transport-linked transmission by integrating questions on mobility and transport use into routine case investigation and by focusing drug-resistant TB surveillance on dense urban routes [2].

In Somalia's fragile, urbanizing context, TB control efforts that neglect public transport are likely missing a critical driver of ongoing transmission. Conceptualizing minibuses and buses as mobile transmission hotspots shifts attention from static facilities to the daily mobility patterns that connect communities. Incorporating transport systems into TB awareness, active case-finding, ventilation improvements, and surveillance is both realistic and urgently needed. Embedding these measures within national TB strategies offers a novel, context-appropriate opportunity to reduce transmission and accelerate progress toward TB control in Somalia.

## CRediT authorship contribution statement

**Mohamed Abdulkadir Hussein:** Conceptualization. **Ilyas Abdullahi Khalif:** Writing – review & editing. **Saida Abdirzak Mohamed:** Writing – original draft. **Sharmake Gaiye Bashir:** Visualization. **Ahmed Abdinasir Abdulle:** Methodology.

## Clinical trial number

Not applicable.

## Consent for publication

Not applicable.

## Ethics approval and consent to participate

Not applicable. This narrative review is based exclusively on previously published literature and does not involve human participants or primary data collection.

## Funding

This research received no external funding.

## Declaration of competing interest

The author declares no competing interests.

## Data Availability

Not applicable.
